# Development and use of three monoclonal antibodies for the detection of rice black-streaked dwarf virus in field plants and planthopper vectors

**DOI:** 10.1186/1743-422X-10-114

**Published:** 2013-04-10

**Authors:** Jianxiang Wu, Yuequn Ni, Huan Liu, Lixia Rao, Yijun Zhou, Xueping Zhou

**Affiliations:** 1 State Key Laboratory of Rice Biology, Institute of Biotechnology, Zhejiang University, Hangzhou, Zhejiang, 310058, China; 2 Institute of Plant Protection, Jiangsu Academy of Agricultural Sciences, Nanjing, 210014, China

**Keywords:** Rice black-streaked dwarf virus, Monoclonal antibody, Small brown planthopper, Antigen-coated-plate enzyme-linked immunosorbent assay, Dot enzyme-linked immunosorbent assay

## Abstract

**Background:**

Rice black-streaked dwarf virus (RBSDV) causes great losses in rice, maize and wheat production in Asian countries. The use of serological methods for RBSDV detection depends on the availability of antibodies. In this study, three highly sensitive and specific murine monoclonal antibodies (MAbs) against RBSDV antigens were produced using crude extracts from tumors of RBSDV-infected maize as the immunogen, and two serological assays, antigen-coated-plate enzyme-linked immunosorbent assay (ACP-ELISA) and dot enzyme-linked immunosorbent assay (dot-ELISA) were developed for RBSDV detection.

**Results:**

All three MAbs reacted strongly and specifically with the crude extracts from RBSDV-infected plant and planthopper tissues. The detection endpoints of three MAbs (12E10, 18F10 and 5G5) in ACP-ELISA were respectively 1:40,960, 1:40,960, 1:81,920 (w/v, g mL^-1^) with the crude extract of infected maize, 1:10,240, 1:20,480, 1:20,480 (w/v, g mL^-1^) with the crude extract of infected rice, 1:5,120, 1:10,240, 1:10,240 (w/v, g mL^-1^) with the crude extract of infected wheat, 1:9,600, 1:9,600, 19,200 (individual planthopper/μL) with the crude extract of infected planthopper. The newly developed ACP-ELISA could detect the virus in the infected maize, wheat, rice tissue crude extracts diluted at 1:81,920, 1:20,480, 1:10,240 (w/v, g mL^-1^), respectively, and in individual viruliferous planthopper extract diluted at 1:19200 (individual planthopper/μL). The dot-ELISA was proved to detect the virus in the infected maize, wheat and rice tissue crude extracts diluted at 1:320 (w/v, g mL^-1^), and in individual viruliferous planthopper extract diluted at 1:1,600 (individual planthopper/μL), respectively. Field plants (915) and planthopper samples (594) from five provinces of China were screened for the presence of RBSDV using the two developed serological assays. The results indicated that 338 of the 915 plant samples and 19 of the 594 planthopper samples were infected by RBSDV.

**Conclusions:**

The newly developed ACP-ELISA and dot-ELISA were highly sensitive and specific to detect RBSDV in field plant and planthopper samples. The field survey demonstrated that RBSDV is widespread in rice, maize and wheat crops in Jiangsu, Zhejiang, Shandong provinces of China.

## Background

Rice black-streaked dwarf virus (RBSDV), a member of the genus *Fijivirus* in the family *Reoviridae*, is mainly transmitted by the small brown planthopper (*Laodelphax striatellus*) in a persistent and propagative manner, but not transmitted via eggs [1-3]. RBSDV naturally infects graminaceous plant species including rice, maize, wheat, barley, and several species of weeds, resulting in rice black-streaked dwarf disease, maize rough dwarf disease and wheat dark-green dwarf disease, respectively, [1,3-5]. The typical symptoms of diseased rice plants include stunting, darkening of leaves and white tumors or black-streaked swellings on stem and abaxial surfaces of leaves, leaf blades and sheaths [6]. The diseased maize plants present stunted, dark green color, white tumors on stem and along the veins on abaxial surface of leaves and leaf sheaths, suppressed flowers and no ears or just nubbins [7]. RBSDV occurs in China, Japan, and other Asian countries and causes severe yield losses in rice, maize, wheat and barley production [3,4,8]. The outbreaks of RBSDV in Japan were recorded in maize during 1957–1961, and in rice and maize during 1965–1967 [1,3]. In China, RBSDV was reported in Zhejiang Province in 1963, and since the early 1990s, the virus caused severe damage in rice in most regions of Zhejiang Province and northern Fujian Province of China [4,8-10]. In recent years, outbreaks of the virus occurred on rice in Jiangsu Province and in maize in some maize-growing areas, causing severe losses in rice and maize production in China. The outbreak of RBSDV generally coincides with a high density of its vector, a high percentage of viruliferous planthoppers in overwintering populations during the most susceptible stage of young crops, and changes in cultivation practices [3,11]. Virions are localized in the phloem and gall tissues in infected plants, viroplasms, virus crystals and tubular structures in both infected plants and planthopper vector cells [3,12,13].

RBSDV virions are non-enveloped, icosahedral, double-shelled particles with 75 to 80 nm in diameter and short surface spikes and contain 10 segments ranging from 1.8 to 4.5 kb of linear double-stranded genomic RNA (designated S1–S10) [9,14]. Most genomic segments only contain one open reading frame (ORF), while S5, S7 and S9 each contain two ORFs [13,15,16]. The core particle of RBSDV consists of four proteins: P1 (RNA-dependent RNA polymerase) encoded by S1, P2 (major core capsid protein) encoded by S2, P3 (putative guanylyltransferase) encoded by S3, and P8 (a minor core capsid protein) encoded by S8 [13,17,18]. The outer layer of the RBSDV particle consists of P4 (outer-shell B-spike protein) encoded by S4 and P10 (outer capsid or coat protein, CP) encoded by S10 [13,19]. The nonstructural protein P6 encoded by S6 functions as a viral RNA silencing suppressor [20]. Both S7 and S9 each encode a nonstructural protein, i.e. P7-1 and P9-1. P7-1 and P9-1 are components of the tubular structures and viroplasms in infected plants and planthopper cells, respectively [13,21].

Currently, some approaches have been used for detection of RBSDV: reverse transcription (RT)-polymerase chain reaction assay (PCR) [22], RT-loop-mediated isothermal amplification assay (RT-LAMP) [22,23], polyclonal antibodies (PAbs)-based indirect enzyme-linked immunosorbent assay (ID-ELISA) [24], and PAbs-based double antibody sandwich enzyme-linked immunosorbent assay (DAS-ELISA) [25]. Among those methods, serological methods are more suitable for routine detection of high throughput samples in the field survey. But, the results of serological methods are dependent on the quality and availability of antibodies. In this study, three highly sensitive and specific murine monoclonal antibodies (MAbs) against RBSDV antigens were produced using the hybridoma technology, and two MAb-based serological methods, antigen-coated-plate enzyme-linked immunosorbent assay (ACP-ELISA) and dot enzyme-linked immunosorbent assay (dot-ELISA) were developed for sensitive and specific detection of RBSDV in field samples. The detection results of field samples by the established two serological methods demonstrated that RBSDV is widespread in rice, maize and wheat crops in Jiangsu, Zhejiang and Shandong provinces of China.

## Results

### Preparation and characterization of MAbs against RBSDV

The crude extract containing RBSDV virions and its non-structure proteins from white tumors of RBSDV-infected maize were used as the immunogen. After the 4^th^ immunization, the spleen cells of the immunized mice were used for hybridoma preparation. Via cell fusion, cell culture, antibody detection and cell cloning, three hybridoma lines (12E10, 18F10 and 5G5) secreting MAbs against RBSDV antigens were obtained and injected intraperitoneally into BALB/c mice to produce ascitic fluids, respectively. The IgG yields of MAbs from ascitic fluids ranged from 2.32 to 7.78 mg mL^-1^ (Table 1). The immunoglobulin classes and subclasses of all three MAbs were isotyped as IgG1, kappa light chain (Table 1). The titers of those three MAbs in ascites determined by an indirect-ELISA ranged from 10^-6^-10^-7^ (Table 1).

**Table 1:** Properties of monoclonal antibodies against RBSDV

**MAbs**	**Isotype**	**Ascites titre**	**IgG yield (mg mL**^**-1**^**)**
12E10	IgG1, κ chain	10^-6 *^	2.32
18F10	IgG1, κ chain	10^-6^	4.51
5G5	IgG1, κ chain	10^-7^	7.78

^*^ The MAb titer was the last dilution that yielded an absorption value above 0.3 at 30 min after adding the substrate at room temperature.

**Figure 1:**
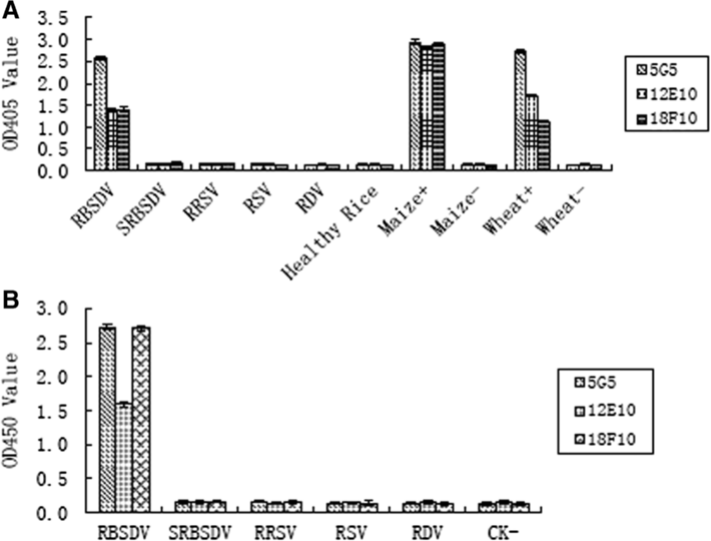
**Specificity analyses of three MAbs by ACP-ELISA. A**: Specificity analyses of three MAbs with five tested plant viruses. RBSDV, SRBSDV, RDV, RRSV and RSV were rice plants infected with RBSDV, SRBSDV, RDV, RRSV and RSV respectively. Healthy rice was a healthy rice plant. Maize+ and Maize– were a RBSDV-infected maize plant and a healthy maize plant, respectively. Wheat+ and Wheat– were a RBSDV-infected wheat plant and a healthy wheat plant, respectively. **B**: Specificity analyses of three MAbs with viruliferous insect vectors. RBSDV, SRBSDV, RRSV, RSV, RDV, CK- were RBSDV-infected small brown planthopper, SRBSDV-infected white-backed planthopper, RRSV-infected brown planthopper, RSV-infected small brown planthopper, RDV-infected leafhopper and non-viruliferous small brown planthopper, respectively.

**Figure 2:**
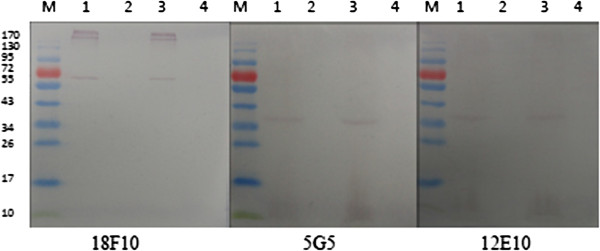
**Western blot analyses of MAbs with RBSDV-infected rice and insect vector tissues.** M was a protein molecular weight marker. Lanes 1 and 2 were RBSDV-infected and healthy rice plants, respectively. Lanes 3 and 4 were viruliferous and non-viruliferous small brown rice planthoppers, respectively.

The ACP-ELISA results showed that the three MAbs (12E10, 18F10and 5G5) reacted strongly with the crude extracts from RBSDV-infected rice, maize and wheat plant tissues, but not with RDV-, SRBSDV-, RRSV-, RSV-infected rice and healthy rice, maize and wheat plant tissues (Figure 1). Western blot was performed to further confirm the specificity of the MAbs. The results revealed that the MAbs (12E10 and 5G5) reacted with a protein of approximately 40 kDa in both crude extracts from RBSDV-infected plant and insect vector tissues, while the MAb 18F10 reacted with a protein of approximately 56 kDa. Based on the molecular weight of the protein, we can suppose that the MAbs 5G5 and 12E10 reacted either with the P7-1 or P9-1 protein, both proteins are components of the tubular structures and viroplasms in infected plants and planthopper cells, respectively [13,21], while the MAb 18F10 was specific to the outer capsid protein (P10). However, the MAb 18F10 also reacted with two other larger protein bands with molecular mass over 170 kDa in both extracts from RBSDV-infected plants and viruliferous vectors (Figure 2). The P10 of RBSDV can self-interact and form oligomeric complexes with yeast two-hybrid system and far-Western analyses [19]. So, the two larger bands may be the results of the reaction of the MAb 18F10 with these oligomeric P10 complexes. No signals were observed with extracts from the healthy plant and non-viruliferous insect vector tissues (Figure 2).

**Figure 3:**
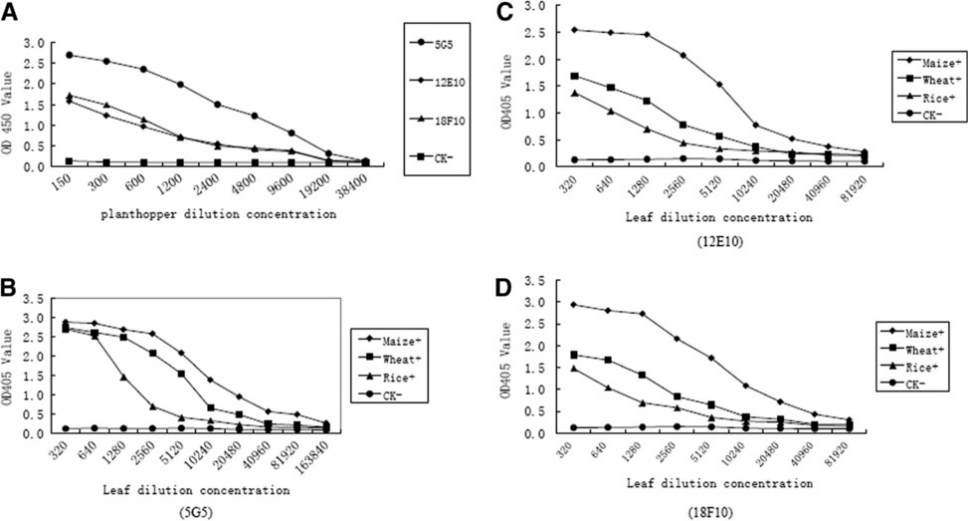
**Sensitivity analyses of three MAbs determined by ACP-ELISA. A**: Sensitivity analyses of three MAbs for RBSDV detection in viruliferous planthopper. Crude extracts from a RBSDV-infected small brown planthopper and a non-viruliferous planthopper (CK^-^) were serial two-fold diluted in 0.05 mol L^-1^ sodium bicarbonate buffer from1:150 to 1:38400 (individual planthopper/μL) and used as coating antigens, respectively. The OD450 value was the mean value obtained from three independent assays at 30 min after adding the substrate at room temperature. **B**, **C**, **D**: Sensitivity analyses of three MAbs for RBSDV detection in infected plant. RBSDV-infected plant tissue extract and healthy tissue extract (CK^-^) were serial two-fold diluted in 0.05 mol L^-1^ sodium bicarbonate buffer from 1:320 to 1:163840 (w/v, g mL^-1^) and used as coating antigens, respectively. The OD405 value was the mean value obtained from three independent assays at 40 min after adding the substrate at room temperature. Rice+, Wheat+ and Maize+ were RBSDV-infected rice, wheat and maize plants, respectively.

The sensitivities of the three MAbs were determined by ACP-ELISA. The endpoint dilutions (i.e., sensitivities) of the three MAbs (12E10, 18F10 and 5G5) in ACP-ELISA were respectively 1:40,960, 1:40,960, 1:81,920 (w/v, g mL^-1^) with the crude extract of infected maize, 1:10,240, 1:20,480, 1:20,480 (w/v, g mL^-1^) with the crude extract of infected rice, 1:5,120, 1:10,240, 1:10,240 (w/v, g mL^-1^) with the crude extract of infected wheat, 1:9,600, 1:9,600, 19,200 (individual planthopper/μL) with the crude extract of infected planthopper (Figure 3). Those above results indicated that all three MAbs have high sensitivity and specificity and can be used for RBSDV detection. The MAb 5G5 was selected for further development of serological assays for the detection of RBSDV because of its higher sensitivity.

### ACP-ELISA for RBSDV detection in plant and planthopper samples

The working dilutions of the MAb 5G5 and the goat anti-mouse IgG conjugated with alkaline phosphatase (AP) or with horseradish peroxidase (HRP) in ACP-ELISA were determined by phalanx tests. The results of the three independent ACP-ELISA assays revealed that RBSDV was readily detected in infected plant tissues when the MAb and the goat anti-mouse IgG conjugated with AP were used at the dilutions of 1:6,000 and 1:7,000, respectively. Meanwhile, RBSDV was readily detected in the planthopper samples when the MAb and the goat anti-mouse IgG conjugated with HRP were used at the dilutions of 1:5,000 and 1:7,000, respectively.

Serial two-fold dilutions with sodium bicarbonate buffer (0.05 mol L^-1^, pH9.6) of RBSDV-infected field plant and planthopper samples were used to determine the sensitivities of the developed ACP-ELISA. The results showed that the ACP-ELISA could detect the virus in infected maize, wheat, rice crude extracts diluted at 1:81,920, 1:20,480, 1:10,240 (w/v, g mL^-1^), respectively, and in individual planthopper crude extract diluted at 1:19200 (individual planthopper/μL) (Figure 3A, B). Specificity analysis of the ACP-ELISA revealed that the ACP-ELISA could highly specifically detect RBSDV in infected plants and viruliferous planthopper samples (Figures 1, 3A, 3B). These results indicated that the ACP-ELISA is sensitive and specific for detecting RBSDV in field samples.

### Dot-ELISA for RBSDV detection in rice and planthopper samples

The optimal working dilutions of the MAb 5G5 and the goat anti-mouse IgG conjugated with -AP or HRP in dot-ELISA were also determined by phalanx tests and the result demonstrated that the optimal dilutions for the detection of the virus in plant samples were 1:5,000 for MAb 5G5 and 1:7,000 for the goat anti-mouse IgG conjugated with AP. To detect the virus in planthopper samples, the optimal dilutions of the MAb and the goat anti-mouse IgG conjugated with HRP were 1:4,000 and 1:6,000, respectively.

**Figure 4:**
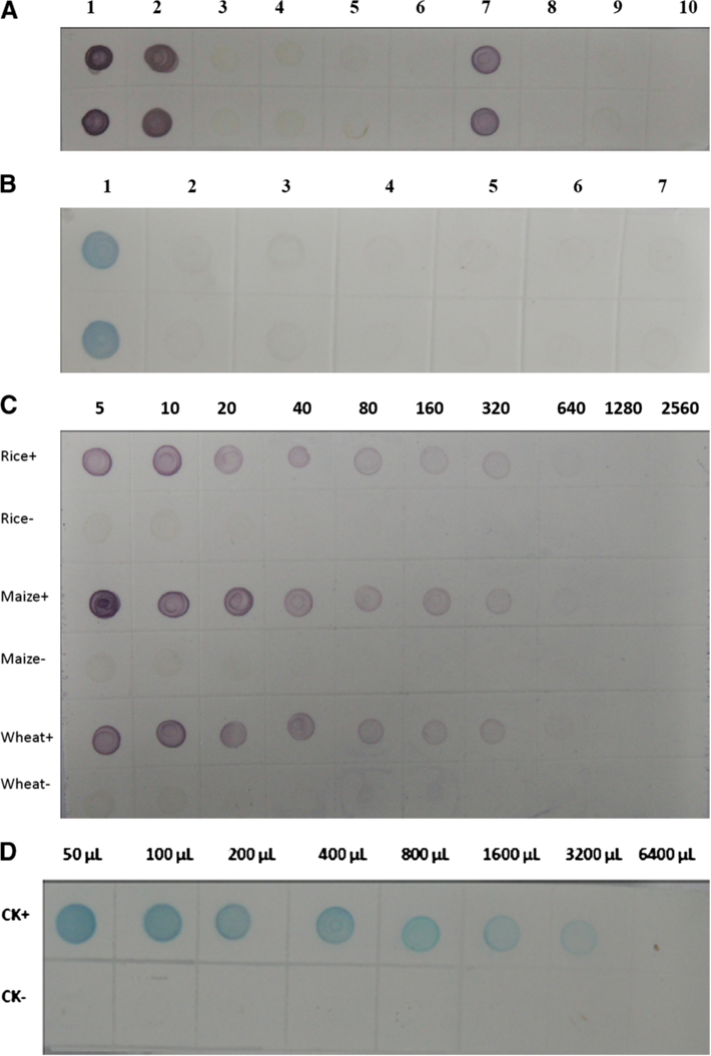
**Specificity (A, B) and sensitivity (C, D) analyses of the dot-ELISA. A**: Specificity of the dot-ELISA for detecting RBSDV in plants. 2, 3, 4, 5 and 6 were rice plants infected with RBSDV, SRBSDV, RSV, RRSV and RDV, respectively; 1 and 7 were RBSDV-infected maize and wheat plants, respectively. 8, 9 and 10 were the healthy rice, wheat and maize, respectively. Up and down dots of the membrane represented repeats of the same sample. **B**: Specificity of the dot-ELISA for detecting RBSDV in insect vectors. 1, 2 and 3 were RBSDV-, RSV-infected and non-viruliferous small brown planthoppers, respectively. 4 and 5 were SRBSDV-infected and non-viruliferous white backed planthoppers, respectively. 6 and 7 were a RRSV-infected brown planthopper and a RDV-infected leafhopper, respectively. Up and down dots of the membrane represented repeats of the same sample. **C**: Sensitivity analyses of the dot-ELISA for detecting RBSDV in infected plants. Lanes Rice+, Maize+ and Wheat+ were RBSDV-infected rice, maize and wheat plants, respectively. Lanes Rice-, Maize- and Wheat- were healthy rice, maize and wheat plants, respectively. Crude extracts from a RBSDV-infected plant and a healthy plant were serial two-fold diluted in 0.01 mol L^-1^ PBS from 1:5 to 1:2560 (w/v, g mL^-1^). **D**: Sensitivity analyses of the dot-ELISA for detecting RBSDV in viruliferous planthopper sample. CK+ and CK- were a viruliferous and a non-viruliferous planthoppers, respectively. Crude extracts from a RBSDV-infected small brown planthopper (CK+) and a non-viruliferous planthopper (CK-) were serial two-fold diluted in 0.01 mol L^-1^ PBS from 1:50 to 1:6400 (individual planthopper/μL).

The specificity of the dot-ELISA was confirmed by a positive reaction of detection with RBSDV-infected plant tissues and negative reactions of detection with RRSV-, SRBSDV-, RDV-, RSV-infected rice plants, or healthy rice, maize and wheat plant tissues. Similarly, the dot-ELISA specifically detected RBSDV in viruliferous small brown planthopper, but negative reactions were obtained with RSV-infected or non-viruliferous small brown planthoppers, SRBSDV-infected or non-viruliferous white backed planthoppers, RRSV-infected brown planthoppers or RDV-infected leafhoppers (Figure 4A, B). The sensitivity analysis indicated that the dot-ELISA could detect the virus in RBSDV-infected maize, wheat and rice tissue crude extracts diluted at 1:320 (w/v, g mL^-1^), and in individual viruliferous planthopper crude extract diluted at 1:1,600 (individual planthopper/μL) (Figure 4C, D).

### Detection of virus in field samples

**Figure 5:**
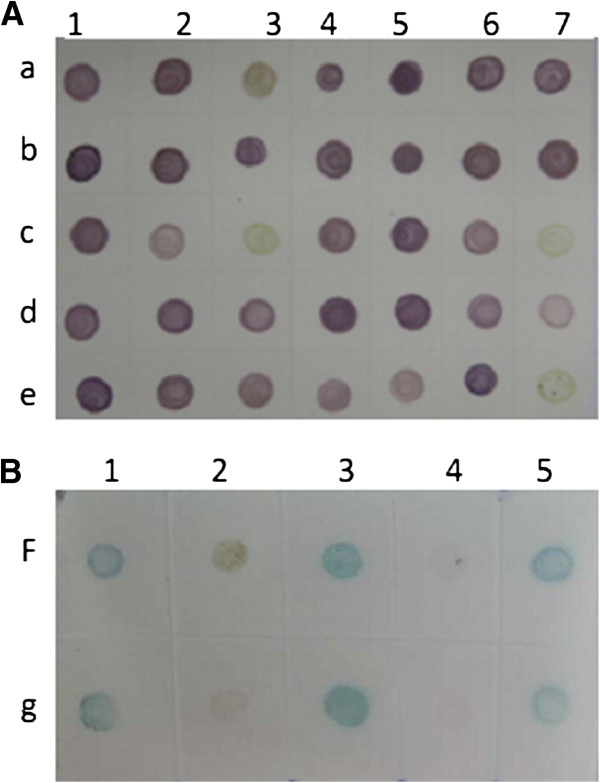
**The detection results of field plant (A) and planthopper (B) samples by the dot-ELISA. A**: The detection results of field plant samples by the dot-ELISA. Lines a and b were maize samples, and a3 and b3 were the negative and positive controls, respectively. Lines c and d were rice samples, and c3 and d3 were the negative and positive controls, respectively. Line e was wheat samples, and e6 and e7 were the positive and negative controls, respectively. Brown or purple color spots indicated positive samples and no color or green spots indicated negative samples. **B**: The detection results of field planthopper samples by the dot-ELISA. f1 and f2 were viruliferous and non-viruliferous small brown planthoppers used as the positive and negative controls, respectively. f3-5 and g1-5 were small brown planthoppers from fields. Blue color spots indicated positive samples and no color or brown spots indicated negative samples.

Field samples including 915 plants and 594 small brown rice planthoppers from Jiangsu, Zhejiang, Shandong, Hainan and Yunnan province of China were screened for the presence of RBSDV and the result was shown in Table 2. A total of 333 plant samples and 18 planthopper samples was tested positive for RBSDV by ACP-ELISA or dot-ELISA (Table 2, Figure 5). The positive results of ACP-ELISA or dot-ELISA were in agreement with that of RT-PCR. However, 5 plants and 1 planthopper samples, which was negative tested by ACP-ELISA and dot-ELISA were positive by RT-PCR (Table 2). RT-PCR amplified-products were sequenced and the resultant nucleotide sequences compared with the RBSDV *CP* gene sequences available in GenBank, and the results showed that the sequences of the amplified products shared 94-99% identity with the RBSDV *CP* gene sequences in GenBank. Those detection results indicate that RBSDV is widespread in Jiangsu, Zhejiang and Shandong provinces of China (Table 2).

**Table 2:** Detection of RBSDV in field samples by ACP-ELISA, dot-ELISA and RT-PCR

**Sample sources**	**RBSDV positive no./rice sample no.**	**RBSDV positive no./maize sample no.**	**RBSDV positive no./wheat sample no.**	**RBSDV positive no./planthopper no.**
Jiangsu Province	245,245,248/250 ^a^	28,28,29/30	11,11,11/12	15,15,16/123
Zhejiang Province	23,23,24/60	16,16,16/21		3,3,3/55
Shandong Province		10,10,10/15		
Hainan Province	0,0,0/320			0,0,0/350
Yunnan Province	0,0,0/151	0,0,0/56		0,0,0/66
Total	268,268,272/781	54,54,55/122	11,11,11/12	18,18,19/594

^a^ 245, 245, 248/250 represent 245, 245, 248 of 250 samples were positive by ACP-ELISA, dot-ELISA and RT-PCR, respectively.

## Discussion

RBSDV causes severe yield losses in rice and maize production in many Asian countries [3,4,8]. After the rice and maize crops are harvested in RBSDV prevalent regions, viruliferous planthopper vector moves first to weeds and then to barley and wheat, where it transmits the virus and oviposits [3]. The following generation acquires the virus on infected plants, and moves to newly planted rice or maize next year to transmit the virus [3]. Hence, it is an effective preventive measure against RBSDV disease to alter the planting time to avoid the viruliferous vectors, while rapid and effective detection of RBSDV in overwintering plants and planthopper populations would permit to select more appropriate planting time. Moreover, rapid detection of RBSDV in field planthopper populations can help to time the spraying of insecticides to efficiently control the viral vectors. So, it is urgent to develop rapid serological methods for RBSDV routine detection in field plant and insect vector.

Virions of fijiviruses are labile and readily break down during the purification processes. In general, most purified RBSDV particles lack the outer capsid and do not to elicit antibodies that could recognize the intact virions when injected into rabbits [26]. Due to the instability of RBSDV virions and their phloem-restriction in host plants, it is very difficult to obtain intact virus particles in sufficient quantity for the preparation of high quality antibodies for the virus detection. The major outer capsid of RBSDV expressed in *Escherichia coli* was used to prepare antiserum, and an ID-ELISA was developed for detecting RBSDV in wheat samples with the antiserum, But, no serological method was developed for RBSDV detection in insect vector [24]. Furthermore, based on the high similarity of outer capsids of Southern rice black-streaked dwarf virus (SRBSDV) and RBSDV, it can be assumed that the antiserum do not distinguish RBSDV from SRBSDV. In Takahashi’s work, serological methods including DAS-ELISA were established and could successfully detect RBSDV in rice samples, but failed to detect RBSDV in vector samples because of nonspecific reaction of the antiserum [25].

In the present study, we used the crude extract from white tumors containing high titre virions and viral non-structure proteins of RBSDV-infected maize plants as the immunogen, which allowed to obtain intact virions and viral non-structure proteins suitable for eliciting antibodies. Three RBSDV-specific MAbs (12E10, 18F10 and 5G5) were then developed. Three MAbs reacted with the crude extracts from the RBSDV-infected plant tissues and viruliferous planthopper, but not with RDV-, SRBSDV-, RRSV- or RSV-infected rice plants, healthy plant tissues or non-viruliferous vectors.

SRBSDV that is a new species in the genus *Fijivirus* in the family *Reoviridae*, is transmitted to rice and maize by the white backed planthopper (*Sogatella furcifera*) in a persistent manner [27,28]. Recently, SRBSDV became one of the most important viruses of rice and maize in Southeast Asian countries [22,27-29]. Both RBSDV and SRBSDV share many similarities in genomic structure and sequence [27-29], virion morphology, serology [27], symptoms and host ranges [22,28]. Hence, in the process of this research, most of the MAbs screened by us can simultaneously react with RBSDV and SRBSDV (data not shown). With many time screenings and selections, we obtained MAbs only reacting with RBSDV but no with SRBSDV.

In order to forecast and control the disease, we have developed two reliable, rapid and efficient serological methods for specific and sensitive detection of RBSDV. Because RBSDV infection is an important problem in cereal crop production in Southeast Asian countries, the serological detection methods developed in this work will support further investigations on the epidemiology of RBSDV and detect the virus more efficiently and economically than the previously available assays.

It is well known that the specificity of MAb is better than PAbs. The dilution endpoints of the DAS-ELISA for RBSDV detection in rice reported by Takahashi et al. [24] and the ID-ELISA for RBSDV detection in wheat reported by Wang et al. [25] both are 1: 1,280 (w/v, g mL^-1^). In this study, the ACP-ELISA could detect RBSDV in infected plant tissue extracts at the dilution of above 1: 10,240 (w/v, g mL^-1^) and in individual viruliferous vector extracts at the dilution of 1:19200 (individual planthopper/μL), while dot-ELISA could detect RBSDV in infected plant tissue extracts at the dilution of 1: 320 (w/v, g mL^-1^) and in individual viruliferous vector extracts at dilution of 1:1,600 (individual planthopper/μL) (Figures 3 and 4). To our knowledge, no serological methods were reported to detect RBSDV in plants and vectors with such lower detection limits.

RT-PCR and serological methods are frequently applied to detect plant RNA viruses. It is well known that the RT-PCR for RNA virus detection is more sensitive than serological methods and our results proved that again. However, the RT-PCR method is complicated, time-consuming, expensive, not suitable for large-scale samples. So serological methods are more often used in the routing detection of plant viruses.

Field plant and small brown rice planthopper samples were screened for the presence of RBSDV with the newly developed serological methods and the detection results showed that RBSDV is widespread in Jiangsu, Zhejiang and Shandong provinces of China (Table 2). We did not detect RBSDV in field samples collected in Hainan and Yunnan provinces of China, but we could not determine whether RBSDV occur in those two provinces and other non-detected provinces of China. Further tests are necessary to confirm the absence of the pathogen in these provinces.

## Materials and methods

### Virus sources and field samples

Crude extracts of the RBSDV-infected maize collected from Jiangsu province were used as antigens for producing MAbs, and crude extracts of the RBSDV-infected rice, maize and wheat samples collected from Jiangsu and Zhejiang provinces were used for screening MAbs. The small brown planthoppers were fed on RBSDV-infected rice plants maintained in a greenhouse to obtain viruses as described previously [25] and used as positive controls for vector screening. Southern rice black-streaked dwarf virus (SRBSDV), Rice dwarf virus (RDV), Rice ragged stunt virus (RRSV) and Rice stripe virus (RSV) were characterized by the author’s laboratory and stored at 4°C.

915 field plant samples showing dwarf symptoms and 594 field small brown rice planthopper samples were collected from Jiangsu, Zhejiang, Shandong, Hainan and Yunnan provinces of China during 2010–2012.

### Preparation of MAbs against RBSDV antigens

The white tumors of RBSDV-infected maize plants from Jiangsu Province were ground to fine powder with a mortar and pestle in liquid nitrogen and homogenized further in PBS in 0.02 mol L^-1^ phosphate buffered saline (PBS, pH7.4) (1 g tumor tissues in 3 mL buffer). The ground homogenate was centrifuged at 5000 × *g* for 5 min at 4°C. The supernatant was used to immunize five eight-week-old BALB/c mice as described previously [30].

Animal experiments were carried out using female BALB/c mice provided by the Shanghai Laboratory Animal Center of the Chinese Academy of Sciences (Certificate of animal quality: Zhong Ke Dong Guan No.003) at the Research Center of the Laboratory of Animal Science, Zhejiang College of Traditional Chinese Medicine, Hangzhou, China. The animal experiments were performed according to the Principles of the Helsinki accords. The experimental protocols were approved by the Animal Ethics Committee of Zhejiang University, Hangzhou, China.

Preparation of hybridomas secreting anti-RBSDV MAbs were performed as described by Wu et al. [31]. Hybridomas were injected into syngeneic BALB/c mice to produce ascitic fluids. An indirect-ELISA using the sap extracted from RBSDV-infected maize plants as the coating antigen was applied to determine the titres of ascitic fluids. The isotypes of MAb were discriminated using a mouse MAbs isotyping kit according to the manufacturer’s instructions (Sigma-Aldrich, St. Louis, MO, USA). The specific and sensitive analyses of MAbs were respectively performed by Western blot and ACP-ELISA as described previously [30,32].

### ACP-ELISA for RBSDV detection

The optimal working concentrations of the MAb and the goat anti-mouse IgG conjugated with AP or with HRP (Sigma-Aldrich) for ACP-ELISA were determined by phalanx tests as described previously (30). Detection of RBSDV in plant tissues or in planthopper vectors was performed following the procedure as described by Shang et al. [30]. The Negative and positive controls consisted of extracts from healthy and RBSDV-infected tissues or from non-viruliferous and viruliferous planthoppers, respectively. The AP conjugate was detected with p-nitrophenyl phosphate (Sigma-Aldrich), while HRP conjugate was detected with 3, 3^′^, 5, 5^′^-Tetramethylbenzidine (TMB) substrate (Promega, Madison, WI, USA). The OD405 or OD450 were measured with a Microplate Reader Model 680 (BIO-RAD, Hercules, CA, USA). Samples were considered to be positive when absorbance values were at least three times greater than the negative controls.

### Dot-ELISA for RBSDV detection

The dot-ELISA Procedures were performed as described previously with slight modifications [30]. Briefly, plant samples were ground to fine powder with a mortar and pestle in liquid nitrogen and homogenized further with a pestle in 0.01 mol L^-1^ PBS (1 g plant tissues in 10 mL PBS). Individual planthopper was placed in a 0.5 mL centrifuge tube containing 50 μL PBS, and mashed with a toothpick. The plant and planthopper homogenates were centrifuged at 5000 × *g* for 3 min at 4°C. Negative and positive controls were the healthy and RBSDV-infected plant tissues or non-viruliferous and viruliferous planthoppers. The supernatants (2 μL) were spotted onto nitrocellulose membranes (Amersham Biosciences, Bucks, UK) and air-dried at room temperature. The nitrocellulose membrane was soaked in PBST containing 5% dried skimmed milk powder (w/v, g mL^-1^) for 30 min to block protein-free areas. Then, the membrane was incubated in suitably diluted or serial two-fold diluted MAb for 1 h at 37°C. After washing four times with PBST and the membrane was incubated in goat anti-mouse IgG conjugated with AP or HRP for another 1 h. Finally, after washing five times with PBST, the membrane was color-developed in NBT/BCIP or TMB substrate solution (Promega, Madison, WI, USA). Positive samples developed either purple or blue color within 10–25 min.

### RT-PCR and sequence analysis

Based on RBSDV *CP* gene (i.e., S10) sequences available in GenBank, the RBSDV S10-specific forward primer (5^′^- ATGGCTGACATAAGACTCGA-3^′^) and reverse primer (5^′^- TCATCTTGTCACTTTGTTTAG -3^′^) (nucleotides 22–1698 of S10) were designed to amplify the RBSDV *CP* gene using a standard RT-PCR protocol described previously [22]. All field plant and planthopper samples were detected for RBSDV by RT-PCR. The PCR products were inserted into T-vector (TaKaRa, Dalian, China), and three independent clones from each sample were sequenced subsequently. All sequences were aligned and their identities were determined by the Clustal W method of the MegAlign procedure supplement within the DNASTAR package (Version 7.0, DNAStar Inc., Madison, WI, USA).

## Competing interests

The authors declare that they have no competing interests.

## Authors’ contributions

JW, YN and HL did most experiments and drafted the manuscript. LR did the RT-PCR for RBSDV detection. XZ and YZ conceived of the study, and participated in its design and coordination. XZ, YZ and JW proof-read and finalized the manuscript. All authors read and approved the final manuscript.
